# T‐cell responses and therapies against SARS‐CoV‐2 infection

**DOI:** 10.1111/imm.13262

**Published:** 2020-10-27

**Authors:** Salman M. Toor, Reem Saleh, Varun Sasidharan Nair, Rowaida Z. Taha, Eyad Elkord

**Affiliations:** ^1^ Qatar Biomedical Research Institute (QBRI) Hamad Bin Khalifa University (HBKU) Qatar Foundation (QF) P.O. Box: 34110 Doha Qatar; ^2^ Biomedical Research Center School of Science, Engineering and Environment University of Salford Manchester UK

**Keywords:** coronavirus, COVID‐19, immune responses, SARS‐CoV‐2, T cells

## Abstract

Coronavirus disease 2019 (COVID‐19) is caused by SARS‐CoV‐2, a novel coronavirus strain. Some studies suggest that COVID‐19 could be an immune‐related disease, and failure of effective immune responses in initial stages of viral infection could contribute to systemic inflammation and tissue damage, leading to worse disease outcomes. T cells can act as a double‐edge sword with both pro‐ and anti‐roles in the progression of COVID‐19. Thus, better understanding of their roles in immune responses to SARS‐CoV‐2 infection is crucial. T cells primarily react to the spike protein on the coronavirus to initiate antiviral immunity; however, T‐cell responses can be suboptimal, impaired or excessive in severe COVID‐19 patients. This review focuses on the multifaceted roles of T cells in COVID‐19 pathogenesis and rationalizes their significance in eliciting appropriate antiviral immune responses in COVID‐19 patients and unexposed individuals. In addition, we summarize the potential therapeutic approaches related to T cells to treat COVID‐19 patients. These include adoptive T‐cell therapies, vaccines activating T‐cell responses, recombinant cytokines, Th1 activators and Th17 blockers, and potential utilization of immune checkpoint inhibitors alone or in combination with anti‐inflammatory drugs to improve antiviral T‐cell responses against SARS‐CoV‐2.

## Graphical abstract

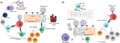

**Abbreviations**
ACE2: angiotensin‐converting enzyme 2
ANG II: angiotensin II
CFR: case fatality rate
COVID‐19: coronavirus disease 2019
CTLs: cytotoxic T lymphocytes
DAMPs: damage‐associated molecular patterns
SARS‐CoV‐2: severe acute respiratory syndrome coronavirus 2
E protein: envelope protein
ICs: immune checkpoints
ICIs: immune checkpoint inhibitors
M protein: membrane protein
N protein: nucleocapsid protein
NLR: neutrophil–lymphocyte ratio
ORFs: open reading frames
PAMPs: viral pathogen‐associated molecular patterns
PRRs: pattern recognition receptors
RLRs: RIG‐I‐like receptors
S protein: spike protein
Tex: T‐cell exhaustion
TLRs: Toll‐like receptors; Tregs, T regulatory cells

## INTRODUCTION

Coronavirus disease 2019 (COVID‐19) is caused by a novel strain of coronavirus, severe acute respiratory syndrome coronavirus 2 (SARS‐CoV‐2).^1^ This latest viral pandemic first surfaced in December 2019 and has since spread aggressively with several millions of confirmed cases, while mortality rates have claimed approximately 3.5% deaths of all infected cases globally.^2^ The close genetic proximity of 2019‐novel coronavirus (nCoV) with SARS coronavirus assisted detection via probing for viral nucleic acid,^3^, ^4^, ^5^ and initial reports revealed pneumonia‐related pathogenesis in COVID‐19 patients.^6^, ^7^ Pre‐existing comorbidities and patient age have since been identified as vital risk factors for case fatality rate (CFR) among others, and multiple organ failure was observed in critically ill patients, who accounted for 5% of 72,314 cases, with 49% CFR in China.^8^ Crucially, there are no proven/approved direct antiviral therapies to treat COVID‐19 patients, and therapeutic strategies revolve around supportive care and treatment of disease symptoms only. However, at present there are more than 2400 clinical trials associated with COVID‐19, investigating potential therapeutic agents and strategies to tackle COVID‐19.^9^

Coronaviruses are known to instigate diseases in humans; OC43, 229E, NL63 and HKU1 classically infect upper respiratory tract, while Middle East respiratory syndrome coronavirus (MERS‐CoV), SARS‐CoV and SARS‐CoV‐2 infect lower respiratory tract.^1^ SARS‐CoV‐2 genome comprises 14 open reading frames (ORFs) encoding 27 proteins, which include major structural proteins: spike (S) protein, membrane (M) protein, envelope (E) protein and nucleocapsid (N) protein.^10^ Angiotensin‐converting enzyme 2 (ACE2) is identified as the main host cell receptor of SARS‐CoV‐2, responsible for allowing viral entry into cells via interactions with its putative ligand, S protein on the coronavirus.^3^ ACE2 is abundantly expressed in lung epithelia;^11^ therefore, lungs are the primary organs affected in COVID‐19.^12^ ACE2 helps to modulate the activity of angiotensin II (ANG II), which elevates blood pressure and promotes inflammation.^13^ SARS‐CoV‐2 has a 10‐ to 20‐fold higher binding affinity with ACE2 compared with other SARS coronaviruses.^14^ Binding of SARS‐CoV‐2 with ACE2 dysregulates ANG II signalling, leading to tissue injury.^15^

SARS‐CoV‐2 infection can initiate a potent immune response, which includes immune activation and antiviral immune responses via helper T cells (Th) and cytotoxic T cells (CTLs),^16^ and induce infected cell death.^17^ However, the transition between innate and adaptive immune responses is crucial in determining disease outcomes of SARS‐CoV‐2 infections; early immune responses primarily have a protective role, whereas dysregulated and exacerbated inflammatory responses can fail in viral clearance and lead to worse disease outcomes.^16^ Accumulation of proinflammatory cytokines, lymphopenia and deviant T‐cell responses present evidence that COVID‐19 might be an immune‐related disease. Moreover, antiviral immunity is mediated by the generation of neutralizing antibodies by plasma cells and CTL‐mediated immunity, which secrete cytokines/effector molecules for killing virus‐infected target cells. M immunoglobulins (IgM) provide primary defence against viral infections, prior to the production of high‐affinity immunoglobulin G (IgG) for lasting systemic immunity, and therefore, their detection can predict exposure times.^18^ However, impaired immune responses, evident from the reduction in lymphocyte levels (lymphopenia) and excessive cytokine release, lead to tissue inflammation and damage in COVID‐19 patients.^19^, ^20^ Lymphopenia and/or T‐cell exhaustion could be one of the major causes of worsened clinical outcomes in COVID‐19 patients, whereas T‐cell‐mediated inflammation and persistent activation of innate immune cells could be contributing factors to lung pathology and secondary complications seen in severe cases.^31^, ^37^, ^69^ Therefore, further understanding of the mechanisms by which the immune response is activated upon SARS‐CoV‐2 infection, roles of innate and adaptive immunities during the course of infection and the contribution of innate and adaptive immune responses to disease recovery and exacerbation is crucial for assigning therapeutic protocols to patients, and the clinical application of pharmacological drugs, which potentially can interfere with the host immunity. The present review focuses on T cells, which are at the forefront of all viral immune responses documented in COVID‐19 patients, to highlight their anti‐ and pro‐roles in disease progression and present plausible targets for therapeutic interventions.

## IMMUNE CELLS IN THE LUNGS OF SARS‐CoV‐2‐INFECTED PATIENTS

Lungs are immensely affected by SARS‐CoV‐2 infection, which leads to lesions with diffused alveolar damage observed in non‐surviving patients.^21^ SARS‐CoV‐2 infects ACE2‐expressing cells in the lungs such as type 2 alveolar cells, leading to the dampening of antiviral IFN responses, while the infiltration of adaptive immune cells in the lungs can lead to heightened inflammatory responses resulting in pulmonary oedema.^22^ Innate immune responses against SARS‐CoV‐2 are initially prompted by lung epithelial cells, alveolar macrophages and neutrophils, which then trigger adaptive immune responses involving T and B lymphocytes.^16^ *Ex vivo* models showed that SARS‐CoV‐2‐infected pneumocytes and alveolar macrophages prompted the release of proinflammatory cytokines and antiviral IFN (type I and III) at low levels.^23^, ^24^

Lung autopsy from a COVID‐19 case provided important insights into the distribution of immune cell infiltrates in the lungs; alveolar exudate showed moderate levels of macrophages and low levels of neutrophils, while interstitial compartment showed infiltration of T cells and monocytes, but not B cells.^25^ Other post‐mortem findings from 38 patients who died with COVID‐19 showed infiltration of macrophages in alveolar lamina and lymphocytes in pulmonary interstitium.^26^

Lymphopenia observed in the circulation of COVID‐19 patients, particularly in those with severe disease, may occur as a result of lymphocyte infiltration and sequestration in the lungs.^27^, ^28^ Moreover, pulmonary influx of immune cells could also potentially justify elevated neutrophil‐to‐lymphocyte ratios recorded in COVID‐19 patients and presented as a biomarker for disease severity and organ failure,^28^ due to imbalances in immune cell infiltrates in the lungs; however, concrete evidence is warranted to support it. Liao *et al*. analysed samples of bronchoalveolar lavage fluid (BALF) from COVID‐19 patients and reported higher levels of macrophages and neutrophils, but lower levels of DCs and CD8^+^ T cells in patients with severe disease compared to those with moderate infection.^29^ Of interest, CD8^+^ T cells in BALFs from patients with severe disease were more proliferative but less clonally expanded compared to those with moderate disease, implicating that CD8^+^ T‐cell responses to SARS‐CoV‐2 in severe cases could be compromised.^29^ Moreover, the authors showed that patients with severe disease have higher expression of activation and migratory genes including *GZMA*, *GZMK*, *ITGA1* and *CXCR6* and higher levels of inflammatory cytokines including IL‐6, IL‐8 and IL‐1β in BALF, reflecting the hyperinflammatory state in the lungs of these patients.^29^ Chua *et al*. performed single‐cell analyses of nasopharyngeal and bronchial samples from COVID‐19 patients and showed that elevated *ACE2* expression is correlated with *IFNG* expression, and showed that these samples exhibit higher expression levels of *CCL2*, *CCL3*, *CCL20*,*CXCL10*,*IL* *8* and *IL* *1B* genes in patients with severe disease, which could promote T‐cell recruitment.^30^ These latter findings demonstrated that epithelial cell/alveolar damage in COVID‐19 patients could be driven by the crosstalk between epithelial and immune cells accompanied by a proinflammatory environment, potentially giving rise to a positive feedback loop that augments inflammation and tissue destruction.^30^ In addition, the massive infiltration of immune cells into the airways of COVID‐19 patients could significantly contribute to acute lung injury and bacterial pneumonia.^10^

## IMMUNE RESPONSES TO SARS‐CoV‐2

**Figure 1:**
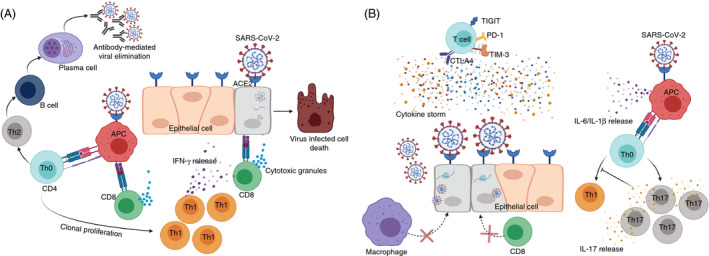
T‐cell responses against SARS‐CoV‐2. SARS‐CoV‐2 recognizes cells expressing ACE2 receptor including epithelial cells and macrophages. In normal immune environment, infected epithelial cells degrade viral particles and present them to cytotoxic CD8^+^ T cells (CTLs). CTLs detect viral protein through classical TCR‐MHC I interaction, release cytotoxic granules, including granzyme B and perforin, and eliminate infected cells. Additionally, macrophages detect SARS‐CoV‐2 via ACE2 receptor and present the virus‐derived peptides to CD4^+^ T cells (Th0) via TCR‐MHC II interaction. Once exposed to antigen, Th0 cells polarize primarily towards Th1, leading to the release of IFN‐γ to eliminate the virus, and Th2 to trigger humoral‐mediated immune responses and antibody secretion against SARS‐CoV‐2 virus (A). In incompetent immune environment, SARS‐CoV‐2 recognizes epithelial cells or macrophages via ACE2 receptor. Viral RNA will replicate by hijacking the host transcriptional machinery. These viral progenies will infect multiple cells leading to tissue damage and further lethal complications. In these circumstances, CD4^+^ and CD8^+^ T cells fail to provide adequate cell/humoral‐mediated immune responses to eliminate viral‐infected cells. On the other hand, Th0 cells are primed towards Th17 phenotype, resulting in the inhibition of Th1‐mediated immune responses (B). In COVID‐19, T cells could be exhausted and could overexpress exhaustion markers including PD‐1, CTLA‐4, TIM‐3 and TIGIT through unknown mechanisms . In severe COVID‐19 cases, the production of cytokines, including IL‐1β, IL‐6, IL‐2, IL‐10 and TNF‐α, is increased leading to the generation of cytokine storm, which induces further unfavourable outcomes and may eventually lead to lymphopenia (B).

The arsenal of innate and adaptive immunity is mostly capable of eliciting adequate antiviral immune responses in mild and moderate cases of COVID‐19 (Figure 1A). Indeed, the co‐ordination between innate and adaptive immune responses during early stages of SARS‐CoV‐2 infection is essential to control viral dissemination.^31^ Moreover, adequate T‐cell counts and sufficient T‐cell activation/clonal expansion have been recorded in COVID‐19 convalescent patients,^32^, ^33^ implying the importance of T‐cell‐mediated immunity in recovery and disease resolution. T‐cell‐dependent protective roles encompass systemic antiviral immune responses and Th‐cell‐mediated activation of B cells, while CTLs have prominent roles in the elimination of virus‐infected cells.^34^ Dendritic cells (DCs) and macrophages can phagocytose virus‐infected cells to initiate T‐cell responses via antigen presentation.^35^ Subsequently, CD4^+^ T cells stimulate B cells for the production of viral‐specific antibodies, and cytotoxic CD8^+^ T cells to target virus‐infected cells. In addition, recognition of viral pathogen‐associated molecular patterns (PAMPs), such as viral RNA or damage‐associated molecular patterns (DAMPs) from host cells, by pattern recognition receptors (PRRs), including RIG‐I‐like receptors (RLRs) and Toll‐like receptors (TLRs), initiates an inflammatory response and leads to elevated secretion of inflammatory cytokines and chemokines, such as interferon‐gamma (IFN‐γ), interleukin (IL)‐6, monocyte chemo‐attractant protein‐1 (MCP1) and C‐X‐C motif chemokine 10 (CXCL10).^19^

Excessive inflammatory innate responses together with impaired adaptive immune responses can cause tissue damage. Infection and lung damage trigger local immune responses resulting in the recruitment of macrophages, which prime adaptive T‐ and B‐cell responses.^1^ Uncontrolled viral infection and high mortality rates in advanced disease and severe cases of COVID‐19 patients resulting from delayed or insufficient activation of T‐cell responses may lead to severe lung damage or systemic inflammation (Figure 1B). However, it is still unclear whether the cytotoxicity observed in severe cases of COVID‐19 infection is caused exclusively by the hyperreactivity of the adaptive immune response or by its suppression.^31^ Moreover, in patients with severe COVID‐19, the total lymphocyte count was dropped to <1.5 × 10^9^/L with overall reduction of 0.31 × 10^9^/L, compared with non‐severe patients.^19^ Lymphopenia has also been observed in non‐surviving COVID‐19 patients, compared with surviving patients.^36^ Therefore, lymphopenia could be considered as a predictive biomarker for the severity of COVID‐19.^19^, ^37^ Additionally, it is important to note that T cells in some COVID‐19 patients could be highly activated,^38^ to mount potent immune responses, or may exhibit functional exhaustion,^39^ indicating weaker immune function, which subsequently results in worse disease outcomes. However, functional studies are necessitated to support these findings.

## SARS‐CoV‐2 RECOGNITION BY T CELLS

Identification of recognizable proteins and epitopes of SARS‐CoV‐2 is crucial to comprehend the reactivity of T cells against SARS‐CoV‐2, and to evaluate T‐cell responses in infected individuals.^40^ Grifoni *et al*. predicted SARS‐CoV‐2 epitopes, which can be recognized by T cells, using bioinformatic approaches such as the Immune Epitope Database and Analysis Resource (IEDB).^40^ Ramaiah *et al*. identified eight T‐cell epitopes distributed across S (n = 2), E (n = 3) and M (n = 3) proteins, which are recognized by all dominant HLA‐DR alleles.^41^ This report suggests that the subunit of vaccine comprising these eight immunodominant epitopes may deliver appropriate T‐cell‐mediated immune responses and development of virus‐specific antibodies.^41^ Additionally, Braun et *al*. demonstrated that 83% of CD4^+^ T cells from SARS‐CoV‐2 patients are spike (S)‐reactive, which could target both N‐ and C‐terminal of S protein and coexpress CD38 and HLA‐DR.^33^ Grifoni *et al*. showed that 11‐27% of total CD4^+^ T cells responded against M, S and N proteins, while CD8^+^ T cells responded against S and M proteins.^40^ Initially, it was reported that S protein of SARS‐CoV‐2 binds to human ACE2‐expressing alveolar pneumocytes.^42^ Later studies confirmed that ACE2 is expressed only on CD169^+^ macrophages in lymph nodes and spleen, and not on T or B lymphocytes.^43^ These macrophages could recognize viral proteins including N and S proteins.^39^ These reports rationalize the importance of T‐cell recognition of SARS‐CoV‐2 proteins to elicit appropriate immune responses to eliminate the virus.

## T‐CELL RESPONSES

### T‐cell responses to SARS‐CoV‐2 in COVID‐19 patients

T‐cell immune responses are considered highly specific and have indispensable roles in eliciting potent antiviral responses (Figure 1A). However, the magnitude of T‐cell responses in being beneficial or harmful for COVID‐19 patients remains unclear due to evidences on their suboptimal, dysfunctional or excessive activity.^34^ The presence of highly activated and fully functional T cells in some COVID‐19 patients has been reported. A study by Weiskopf *et al*. demonstrated that SARS‐CoV‐2‐specific CD4^+^ and CD8^+^ T cells are evident in the peripheral blood of COVID‐19 in the first 2 weeks after onset of symptoms.^44^ Additionally, authors demonstrated that the majority of SARS‐CoV‐2‐specific CD4^+^ T cells exhibited a central memory phenotype with a dominant production of Th1 cytokines, while CD8^+^ T cells had a more effector phenotype with high levels of perforin expression.^44^ In another study, it was reported that levels of CD38‐ and HLA‐DR‐expressing memory CD4^+^ T cells and CD8^+^ T cells, and CD4^+^PD‐1^+^ memory T cells were higher in a proportion of patients with severe COVID‐19, compared with healthy individuals.^28^, ^38^ Importantly, the functionality of memory CD8^+^ T cells and CD8^+^PD‐1^+^CD38^+^ T cells in severe COVID‐19 patients was demonstrated by the high expression levels of perforin and granzyme B, compared with those from healthy controls,^33^ implicating that PD‐1 and CD38 act as activation T‐cell markers in COVID‐19 cases. Moreover, around two‐thirds of COVID‐19 patients showed highly proliferative Ki‐67^+^, non‐naïve CD4^+^ and CD8^+^ T cells, with similar levels of activation in both T‐cell subsets.^38^ Notably, both of these studies reported heterogeneity in T‐cell activation in COVID‐19 patients and provided evidence that both CD4^+^ and CD8^+^ T cells are capable of mounting potent immune responses with potential emergence of impaired or excessive T‐cell responses.

T cells are elevated in patients with mild COVID‐19, creating a robust antiviral immune response.^29^. In particular, CD8^+^ T cells express higher levels of cytotoxic molecules such as granzyme A and FAS ligand, which are beneficial in eliminating virus‐infected cells.^29^ However, in severe disease cases, the aforementioned cytotoxic molecules were reduced due to the reduction in the proportion of CTLs.^29^ Studies on phenotypical characterization of CD4^+^ and CD8^+^ T cells showed that similar percentages of naïve, central memory and effector CD4^+^ T cells were detected in COVID‐19 patients and healthy controls. However, the percentages of both naïve and central memory CD8^+^ T cells were lower in COVID‐19 patients ^45^. Additionally, percentages of terminally differentiated effector CD4^+^ and CD8^+^ T cells were higher in COVID‐19 patients, compared with healthy controls.^45^ Notably, the levels of regulatory T cells (Tregs) in severe COVID‐19 patients were lower than those of mild cases.^46^ Together, these dysregulations in the balance of T‐cell subsets, including Tregs, Th1, naïve and memory T cells, could contribute to severe inflammatory conditions, and perhaps lead to COVID‐19 relapse.^47^, ^48^

Apart from conventional T cells, Rijkers *et al*. reported that a special group of γδ T cells, Vγ9Vδ2, were markedly decreased in non‐surviving patients, compared with healthy or surviving COVID‐19 patients at the time of hospital admission.^49^ Altogether, these reports rationalize the significance of T cells to elicit appropriate antiviral responses against SARS‐CoV‐2 infection and present them as potential prognostic biomarkers.

Although early antiviral responses mediated by CD4^+^ and CD8^+^ T cells are most likely to be protective, the effectual innate immune evasion capabilities of SARS‐CoV‐2 make T cells difficult to generate efficient antiviral responses by limiting IFN type I and III responses.^50^ Importantly, it has also been reported that during the course of COVID‐19 progression, late T‐cell responses might amplify other pathological disease outcomes.^51^ Several clinical trials (NCT04340921, NCT04410432, NCT04403880, NCT04401436, NCT04351711, NCT04403061 and NCT04365166) have been registered to characterize T‐cell phenotypes and measure T‐cell‐derived cytokines in COVID‐19 patients with different disease phases (symptomatic, mild and severe) in order to evaluate the contribution of T‐cell‐mediated immunity to disease outcomes, including secondary complications such as myocarditis.

### T‐cell responses in COVID‐19 convalescent patients and unexposed healthy individuals

Restoration of T‐cell counts in recovered patients could provide important insights into the role of T cells in antiviral responses. SARS‐CoV‐2‐specific T cells were present in more than 70% of COVID‐19 convalescent patients.^40^ Grifoni *et al*. quantified the SARS‐CoV‐2‐specific CD4^+^ and CD8^+^ T cells and found that 100% CD4^+^ T cells and 70% CD8^+^ T cells have SARS‐CoV‐2 spike‐specific responses in recovered patients.^40^ Functional assays confirmed that CD4^+^ T cells appeared as Th1 phenotype and produced substantial amount of IFN‐γ and expressed lower levels of IL‐4, IL‐13, IL‐5, or IL‐17A against S protein.^40^ Likewise, the majority of SARS‐CoV‐2 spike‐specific CD8^+^ T cells produced IFN‐γ and the vast majority of IFN‐γ^+^CD8^+^ T cells also coexpressed granzyme B and tumour necrosis factor‐alpha (TNF‐α).^40^ These data suggest that the majority of CD4^+^ and CD8^+^ T cells in recovered patients generated substantial antiviral immune responses against S protein, implicating the importance of functional T cells in viral clearance and recovery. Moreover, these data rationalize the significance of utilizing SARS‐CoV‐2 S protein as a key candidate for vaccine generation.

Incidentally, SARS‐CoV‐2‐reactive T cells have also been found in unexposed healthy individuals,^33^, ^40^ possibly due to previous exposure, residual immunity and/or close genetic proximity of SARS‐CoV‐2 with other coronaviruses. A study on T‐cell responses against S and M proteins of SARS‐CoV‐2 in unexposed individuals and asymptomatic or mild/severe COVID‐19 convalescent patients showed that highest T‐cell responses were observed in severe convalescent patients and lowest in asymptomatic and unexposed individuals.^52^ Furthermore, compared with antibody‐sero‐positive individuals, detectable T‐cell responses were observed in antibody‐sero‐negative individuals, albeit at lesser frequency.^52^ Finally, authors concluded that asymptomatic/mild COVID‐19 convalescent patients could generate a robust and durable memory T‐cell responses to prevent recurrent infections, even though in the absence of concurrent humoral responses.^52^ In concordance with this study, it has been reported that asymptomatic patients show weaker immune responses, compared with symptomatic individuals, and considerable percentage of symptomatic patients showed reduced amount of neutralizing antibody at the early stages of convalescence.^53^ COVID‐19 convalescent patients also showed a strong correlation with neutralizing antibody titre against human ACE2 and virus‐specific T‐cell counts in 2 weeks post‐hospital discharge.^47^

## COVID‐19 PATHOPHYSIOLOGICAL AND IMMUNOLOGICAL FEATURES

Acute respiratory distress syndrome (ARDS) is the primary complication of SARS‐CoV‐2 infection.^1^, ^54^ SARS‐CoV‐2 infection causes diffused alveolar damage in the lung and damage in the hyaline membrane in alveoli, leading to interstitial widening and oedema and resulting in difficulty in breathing.^55^ ARDS causes respiratory failure that caused 70% , while sepsis contributed to approximately 28% of COVID‐19‐related fatalities.^1^ Moreover, about 17% of the patients recovering from COVID‐19 disease have fibrous stripes, indicating that the lesions are developed during the chronic pulmonary inflammation.^56^

Inflammation of pulmonary endothelial cells (endotheliitis) is one of the contributing factors to the initiation and progression of ARDS by altering the integrity and function of the vascular barrier.^57^ The mechanisms underlying these pathological changes are associated with increased vascular permeability, binding of SARS‐CoV‐2 virus to ACE2 receptors, recruitment of activated neutrophils, macrophages and other immune cells, and increased production of inflammatory cytokines ^57^, ^58^. Some of these cytokines further amplify the inflammatory loop and induce the recruitment of more inflammatory cells, while other cytokines initiate and activate the coagulation cascade.^57^ The resultant intense immune response is extensively documented in ARDS affecting lungs, but leads to multiorgan dysfunction (MODS) failure via tissue damage and ultimately death in severe SARS‐CoV‐2 infections.

A wide range of secondary complications have been associated with SARS‐CoV‐2 infection, including venous thromboembolism,^59^ cardiovascular complications,^60^ acute liver and kidney injury,^61^, ^62^ neurological complications,^24^, ^53^, ^63^ immune thrombocytopenia,^64^, ^65^ secondary infection,^19^, ^66^ septic shock,^19^, ^67^ acute respiratory failure,^62^, ^68^, ^69^ disseminated intravascular coagulation (DIC)^70^, ^71^ and cytokine release syndrome.^72^, ^73^, ^74^ These secondary complications may result from uncontrolled viral dissemination leading to systemic cytokine storm and excessive inflammation.^75^. In this section, we focus primarily on disease mechanisms or complications of COVID‐19 caused either by hyperactivated T cells or by insufficient T‐cell responses.

### Cytokine storm

‘Cytokine storm’ refers to the plethora of proinflammatory cytokines and chemokines detected in various pathological conditions, and is one of the key pathological features observed in SARS‐CoV‐2‐infected patients.^36^, ^73^ High cytokine levels have been recorded in critically ill COVID‐19 patients.^19^ Various immune cell types, including macrophages, neutrophils, DCs, and NK, B and T cells, can contribute to cytokine storm and the hyperactivation state of the inflammatory response in COVID‐19 patients.^20^

TNF‐α, IL‐6 and IL‐1β, principally released by innate immune cells, can be one of the major driving forces for cytokine release syndrome and severe systemic inflammatory responses in patients with advanced stages of SARS‐CoV‐2 infection,^76^, ^77^, ^78^, ^79^ and some of them could be one of the underlying mechanisms responsible for lymphopenia and/or inadequate Th1 responses in these patients.^39^, ^80^ It was also reported that elevated serum levels of TNF‐α and IL‐6 were negatively correlated with the total T‐cell count in severe cases of COVID‐19, indicating the potential involvement of these cytokines in lymphopenia and T‐cell loss.^39^ Conversely, patients in the recovery phase had a marked reduction in serum levels of the aforementioned cytokines and showed a restoration of T‐cell count.^39^ In light of these findings, it was proposed that IL‐6 blockers, such as sarilumab, siltuximab and tocilizumab, and IL‐1β receptor blocker can have therapeutic efficacy in treating severe cases of COVID‐19 patients to resolve hyperinflammation and control the propagation of the pathological immune response to virus infection.^78^, ^81^, ^82^ However, the therapeutic efficacy and safety of IL‐6 and IL‐1β blockers in COVID‐19 patients are currently under clinical investigations.^9^

Elevated levels of chemokines and cytokines, such as CCL2/3/5, CXCL8/9/10 and IFN‐γ, TNF‐α, IL‐1β, IL‐1RA, IL‐6, IL‐7, IL‐8, IL‐12, IL‐33, granulocyte/granulocyte‐macrophage colony‐stimulating factors (G‐CSF and GM‐CSF), vascular endothelial growth factor A (VEGFA) and platelet derived growth factor subunit B (PDGFB), facilitate the recruitment of other leukocytes to tissues and promote effector functions leading to severe ARDS and tissue damage (Figure 1B).^77^, ^83^ Furthermore, Th17‐derived cytokines have been implicated in the excessive lung pathology observed in ARDS patients,^84^ potentiating their contribution to ARDS in COVID‐19 patients,^45^ and rationalizing the potential therapeutic benefits of targeting Th17 cytokines (discussed in the next Section).

### T‐cell activation or exhaustion

T‐cell‐mediated adaptive immune responses are essential for viral clearance and long‐term antiviral immunity, but may contribute to cytokine storm and could be compromised in severe cases of COVID‐19 patients due to T‐cell exhaustion.^31^, ^39^, ^75^ Thus, T cells execute antiviral activities or contribute to tissue inflammation or damage, depending on the host immune response activation status.^85^ It has been suggested that the signature cytokine storm of COVID‐19 may promote Th17‐induced vascular leakage and permeability.^86^ Elevated cytokine levels lead to autoimmune and inflammatory responses that influence the development of ARDS. Activated CD8^+^, Th1, Th17, NK and NKT cells together with other innate immune cells secrete additional cytokines to target virus‐infected cells, and their overstimulation together with effector innate immune cells may lead to tissue damage.^84^

T cells can express high levels of inhibitory immune checkpoints such as PD‐1, TIM‐3, CTLA‐4 and TIGIT upon activation.^87^ On the other hand, sustained expression of inhibitory immune checkpoints in response to persistent antigen stimulation can lead to progressive loss of effector functions; a state known as T‐cell exhaustion and has been observed in severe viral infections.^88^, ^89^ There is evidence suggesting that T cells in COVID‐19 patients could have an exhausted phenotype, indicated by the overexpression of inhibitory immune checkpoints and reduced expression levels of genes encoding cytokines and cytolytic molecules.^39^, ^90^, ^91^ However, the impact of immune checkpoint overexpression on T‐cell effector function, and T‐cell capacity of proliferation and viral clearance has not been elucidated in COVID‐19 patients.

High proportions of both activated CD4^+^HLA‐DR^+^CD38^+^ T cells and CD4^+^PD‐1^+^CD57^+^ exhausted or senescent T cells were detected in COVID‐19 patients, compared with healthy control patients.^45^ Zheng *et al*. demonstrated that CD4^+^ T cells with low levels of IFN‐γ, IL‐2 and TNF‐α were higher in severe COVID‐19 patients, compared with healthy controls and mild patients.^90^ Moreover, CD8^+^ T cells expressing high levels of PD‐1, CTLA‐4, TIGIT, granzyme B and perforin were increased in the severe group, compared with the mild group.^90^ These data suggest that SARS‐CoV‐2 infection may lead to the functional impairment in CD4^+^ T cells and uphold excessive activation of CD8^+^ T cells. Moreover, the frequency of CD8^+^PD‐1^+^CTLA‐4^+^TIGIT^+^ T cells in the circulation of patients with severe COVID‐19 infection was higher than that of mild cases,^90^ suggesting the potential of their exhaustion (Figure 1B). In another study, it was reported that NK cells and CD8^+^ T cells of SARS‐CoV‐2 patients have higher expression of NK cell inhibitory receptor, NKG2A, characterized by reduced intracellular levels of CD107a (degranulation marker), IL‐2, IFN‐γ, TNF‐α and granzyme B, indicating the functional impairment of NK and CD8^+^ T cells in these patients.^91^ While these studies provide evidence of immune checkpoint overexpression on T cells from COVID‐19 patients, particularly those with severe cases, further studies are warranted to determine whether this occurs as a result of T‐cell activation or exhaustion.

## THERAPEUTIC APPROACHES TO IMPROVE T‐CELL RESPONSES IN COVID‐19 PATIENTS

There are potential therapeutic approaches, which can be used for COVID‐19 treatment, such as antivirals, antibodies targeting SARS‐CoV‐2 proteins and antibodies from recovered COVID‐19 patients. However, in this review we will focus on therapeutic approaches, which are directly or indirectly related to T‐cell responses, T‐cell‐derived cytokines and the potential combined therapies aimed at improving virus‐specific T‐cell responses, Th1 responses, expanding T‐cell counts, reversing T‐cell exhaustion and settling inflammation. Clinical trials that have been designed to assess the safety and efficacy of several therapeutic strategies aiming to improve T‐cell responses against SARS‐CoV‐2 infection are listed in Table 1.

**Table 1:** Therapeutic strategies to improve antiviral T‐cell responses and resolve systemic inflammation in COVID‐19.

Therapeutic strategy	Drug/inhibitor	Clinical trial number	Potential benefits	Ref.
Adoptive T‐cell transfer (SARS‐CoV‐2‐reactive T cells) SARS‐CoV‐2‐reactive T‐cell‐derived IFN‐γ exosomes	‐	NCT04351659 NCT04401410 NCT04389385	Improved specific antiviral T‐cell responses against SARS‐CoV‐2	^9^
Viral vector‐based vaccines mRNA‐based and DNA‐based vaccines	‐	NCT04313127 NCT04398147 NCT04341389 NCT04276896 NCT04283461 NCT04336410	Improved specific antiviral T‐cell responses against SARS‐CoV‐2 and production of IFN‐γ	9, 101
Recombinant IL‐7	CYT107	NCT04407689 NCT04379076 NCT04426201	Restored T‐cell count and reverse lymphopenia Enhanced TCR repertoire diversity and generation of memory CD8+ T cells Improved trafficking of T cells to infection site	^9^
Low dose of recombinant IL‐2	ILT101	NCT04357444	Expansion/activation of Tregs to control excessive inflammation Expansion of other T‐cell subsets, including effector cells	^9^
Th1 activators	IFN‐β1b (Ziferon)	NCT04343768	Improved symptoms Activated Th1 response Viral clearance Ameliorated inflammation induced by cytokine storm	^113^
Th17 blockers	Anti‐IL‐17, IL‐17R and anti‐IL‐23	N/A		^114^
JAK2 inhibitor	Fedratinib	N/A		^114^
ICIs	Anti‐PD‐1 (pembrolizumab or nivolumab)	NCT04268537 NCT04333914 NCT04356508 NCT04413838	Reversal of T‐cell exhaustion Restored effector T‐cell function	9, 120

### Adoptive T‐cell therapy against COVID‐19

**Figure 2:**
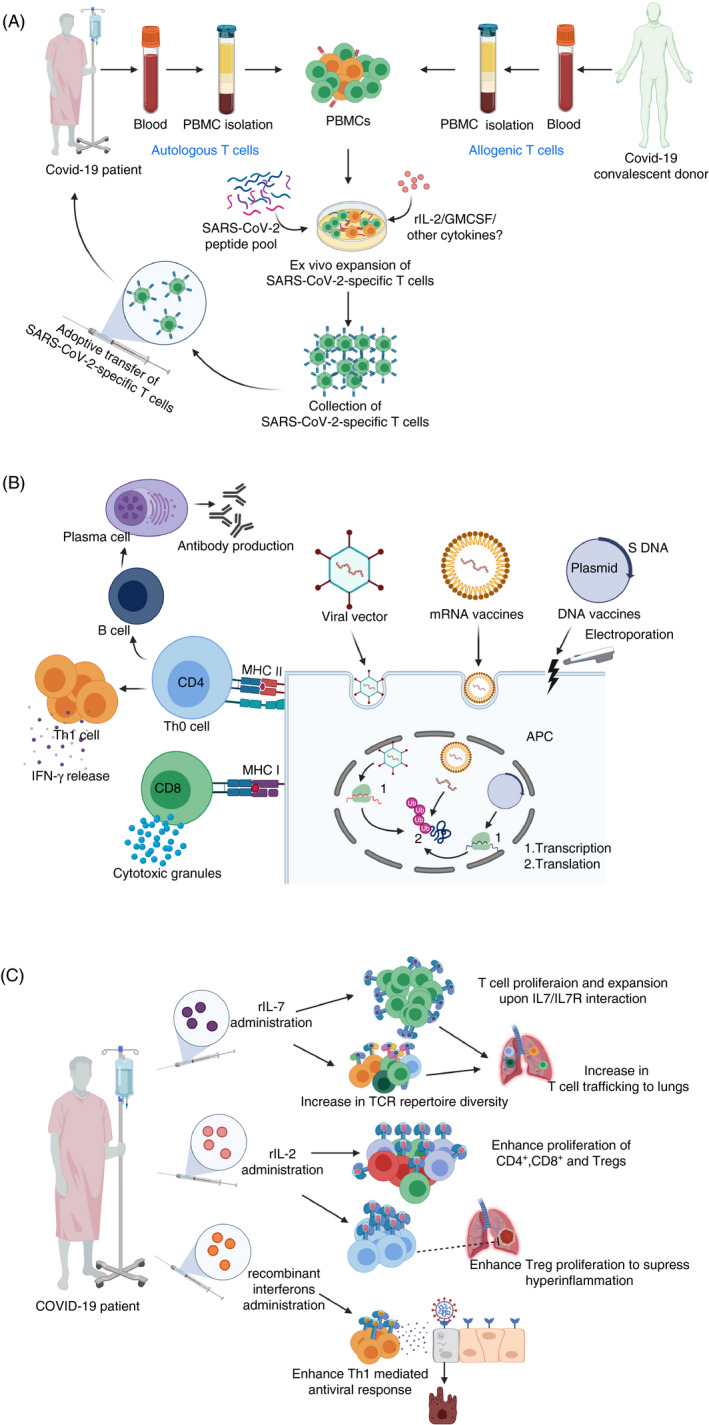
Therapeutic strategies to restore SARS‐CoV‐2‐specific T‐cell immune responses. Autologous (from a COVID‐19 patient) or allogeneic (from a recovered patient), peripheral blood mononuclear cells (PBMCs) can be cultured in the presence of SARS‐CoV‐2‐derived peptides, IL‐2, GM‐CSF or possibly other cytokines to enhance the function of antigen‐presenting cells, enrich T cells and enhance the generation of activated viral‐specific T cells, which can be infused back into a COVID‐19 patient. This could restore effective antiviral T‐cell immunity, and result in beneficial clinical outcomes (A). The use of viral vector‐based, mRNA‐based or DNA‐based vaccines expressing the S protein of SARS‐CoV‐2 can lead to the activation of virus‐specific CD4^+^ and CD8^+^ T cells via antigen‐presenting cells, followed by the activation of B cells and secretion of antibodies by plasma cells (B). In COVID‐19 patients, the administration of recombinant IL‐7 could enhance T‐cell receptor repertoire diversity, promote the capacity of T‐cell trafficking to the lungs and alleviate lymphopenia leading to enhanced antiviral immune response. Low‐dose recombinant IL‐2 could control ARDS and excessive inflammation by expanding and activating Tregs, and possibly increase the level of effector CD4^+^ and CD8^+^ T cells as they express IL‐2 receptor. Additionally, administration of interferons could also help in viral clearance and improving antiviral T‐cell responses (C).

Building on decades of old knowledge of adoptive T‐cell therapies in various pathological conditions including viral infections, utilization of virus‐specific T cells against SARS‐CoV‐2 seems a logical therapeutic approach for treating COVID‐19. Autologous or allogeneic viral‐specific T cells can be expanded *in vitro* and infused to restore effective antiviral immunity, and have shown efficacy in treating various viral infections.^92^ SARS‐CoV‐2‐specific T cells can be isolated from circulation of convalescent donors and expanded using SARS‐CoV‐2‐derived peptides and exploited for treating severe cases of COVID‐19 (Figure 2A). However, the efficacy, treatment‐related toxicities and challenges associated with utilization of adoptive T‐cell therapy have limited its use in COVID‐19. Importantly, it is not possible to utilize unmatched allogenic T cells due to the genetic restrictions (HLA class I), and *in vitro*‐expanded T cells by prolonged stimulation to achieve required cell yields could exhibit functional exhaustion or transferred T cells could in turn contribute to cytokine storm, leading to disease complications of COVID‐19.^93^ However, a clinical trial based on novel adoptive T‐cell therapy for COVID‐19 is ongoing (NCT04351659), while another planned clinical trial will adapt an innovative approach of utilizing already‐collected SARS‐CoV‐2‐specific T cells from recovered patients to treat COVID‐19 patients with high risk of respiratory failure (NCT04401410) (Table 1). Another therapeutic strategy that has been proposed to improve antiviral Th1 responses is based on the use of SARS‐CoV‐2‐specific T cells with IFN‐γ exosomes; clinical trial has been registered to test efficacy in COVID‐19 patients (NCT04389385) (Table 1).

### SARS‐CoV‐2 vaccines activating T‐cell responses

The rapid availability of complete genome of SARS‐CoV‐2 led to identification of numerous candidates for vaccine development, and the role of T cells in vaccine‐mediated immunity is unprecedented due to the generation of effector and memory T cells following stimulation of naïve T cells.^93^ Interestingly, SARS‐CoV‐2‐reactive T cells were found in around 40‐60% of unexposed healthy individuals,^33^ which suggested the presence of cross‐reactive T cells and passive immunity in general populations. Successful vaccines should generate SARS‐CoV‐2‐reactive T cells with high specificity for potent immune responses devoid of the undesired effects of inflammation or inception of disease.

S protein of SARS‐CoV‐2 is identified as the most suitable target for vaccine development to trigger virus‐specific T‐cell responses and humoral immune responses.^94^ The adenovirus‐based viral vector vaccine expressing S protein, adenovirus type‐5 (Ad5‐nCoV), is among the pioneering viral vaccines designed to tackle COVID‐19^95^ (Figure 2B). Zhu *et al*. reported the safety and tolerability of administering a single dose of adenovirus type 5‐vectored COVID‐19 (Ad5‐nCoV) vaccine in healthy individuals (NCT04313127), and its success in producing specific antiviral T‐cell and humoral immune responses after 2 weeks of administration.^96^ Of note, there was a marked increase in the production of IFN‐γ, TNF‐α and IL‐2 by CD4^+^ and CD8^+^ T cells post‐vaccination.^96^ Two additional clinical trials testing Ad5‐vectored COVID‐19 vaccines are also registered (NCT04398147 and NCT04341389). In addition, vaccines based on DCs or artificial APCs modified with lentiviral vector expressing synthetic SARS‐CoV‐2 proteins will be also tested in clinical trials (NCT04276896 and NCT04299724)^95^ (Table 1).

mRNA‐based vaccine, encoding SARS‐CoV‐2 antigen (S protein) to be administered via liposomal delivery system, has been developed by Moderna, and it is under clinical investigation (NCT04283461) to assess its safety and efficacy in COVID‐19 patients^9^, ^97^ (Figure 2B, Table 1). The advantages of using mRNA vaccines include the mimicking of natural viral infection and their safe use as they contain only a short synthetic version of the viral mRNA, which encodes only the antigen protein, and it cannot be integrated into the host chromosomes. Hence, mRNA‐based vaccines are safer than protein‐based vaccines as there are no potential risks associated with virus reactivation. Studies from animal models showed the potential benefits of using newly developed DNA‐based vaccines targeting SARS‐CoV‐2 antigens in inducing T‐cell responses characterized by levels of IFN‐γ secreted by CD8^+^ and CD4^+^ T cells, along with humoral immune responses^98^, ^99^ (Figure 2B, Table 1). The safety, clinical efficacy and immunogenicity of a DNA vaccine named INO‐4800, carrying the DNA fragment of the S protein, will be investigated in phase 1/2 clinical trial, involving healthy individuals, in China and South Korea (NCT04336410)^100^, ^101^

### IL‐7 and Low dose of IL‐2 to restore the repertoire of T cells

IL‐7 can enhance T‐cell receptor repertoire diversity,^102^ promote the capacity of T‐cell trafficking to infection sites,^103^ induce the proliferation of naïve and memory T cells and increase the circulating pool (CD4^+^ and CD8^+^ T cells).^104^, ^105^ On these grounds, clinical trials to evaluate the efficacy of recombinant IL‐7 to restore lymphocyte counts in COVID‐19 patients have been registered (NCT04407689, NCT04379076 and NCT04426201) (Figure 2C, Table 1). The administration of low‐dose recombinant IL‐2 in COVID‐19 patients has been proposed as an alternative therapeutic strategy to control ARDS and excessive inflammation by expanding and activating Tregs; a clinical trial has been registered (NCT04357444) (Figure 2C, Table 1). IL‐2 is a growth factor and important cytokine for the survival and proliferation of Tregs and T effector cells,^106^, ^107^ and therefore administrating low doses of recombinant IL‐2 in COVID‐19 patients should resolve lymphopenia and restore normal T‐cell counts. The safety of using low dose of recombinant IL‐2 and its efficacy in expanding and activating Tregs in patients with autoimmune diseases have been reported.^108^, ^109^, ^110^

### Th1 activators (IFNs) and Th17 blockers (anti‐IL‐17, anti‐IL‐17R and anti‐IL‐23)

It has been suggested that activated potent adaptive immune responses during early disease stages may correlate with improved clinical outcomes. One case report by Thevarajan *et al*. showed evidence suggesting that the recruitment of immune cells, including activated CD4^+^ and CD8^+^ T cells, follicular T helper cells and plasma cells secreting IgG and IgM antibodies against SARS‐CoV‐2 virus in the blood of a patient with symptomatic non‐severe case of COVID‐19 infection, precedes disease resolution and recovery.^111^. Furthermore, low serum levels of proinflammatory cytokines and chemokines, such as IFN‐γ, IL‐6, IL‐8 and MCP‐1, were observed in the patient, when symptoms were evident,^111^ suggesting a potential relationship between symptom development and inadequate immune responses. Thus, sufficient activation of immune response, in particular adaptive immune response, for adequate cytokine production, including IFN‐α/β/γ, IL‐12 and IL‐15, is crucial for viral clearance and symptomatic recovery.^112^

A study by Kuppalli *et al*. demonstrated that severe COVID‐19 disease is associated with elevated levels of IL‐6, reduced levels of CD8^+^ T cells, suppressed Th1 antiviral responses and increased levels of IL‐10, suggesting that cytokine storm together with suppressed Th1 antiviral adaptive responses may lead to severe COVID‐19.^80^ On this basis, a randomized controlled clinical trial to test the clinical efficacy and safety of the administration of IFN‐β1 and IFN‐β2 in moderate‐to‐severe COVID‐19 patients has been designed (NCT04343768) (Figure 2C, Table 1).^113^

Targeting Th17 responses could offer another therapeutic strategy for treating severe COVID‐19 patients, with a predominant Th17 immune profiles and complications associated with cytokine storm.^86^, ^114^ IL‐17A can augment the release of proinflammatory cytokines by innate immune cells, including GM‐CSF, IL‐23, IL‐1β and IL‐6, and propagate lung pathology during ARDS,^115^ and its blockade was beneficial in ameliorating lung inflammation in murine models^116^ and myocarditis,^117^ which is one of the major causes of high mortality rates in COVID‐19 patients.^86^ Thus, targeting Th17 responses via antibodies targeting IL‐17, IL‐17R and IL‐12/23p40 or Janus kinase (JAK2)‐specific inhibitor (which does not interrupt IFN signalling) could be used as future therapeutic approaches to minimize immunopathology caused by cytokine storm without interfering with Th1 response in cases of severe COVID‐19^114^ (Table 1).

### Immune checkpoint inhibitors

As mentioned above, SARS‐CoV‐2 infection may induce T‐cell exhaustion by increasing the expression of inhibitory immune checkpoints (ICs),^39^, ^90^, ^91^ leading to loss of effector T‐cell functions including viral clearance. Importantly, elevated PD‐1 and TIM‐3 expressions were recorded in COVID‐19 patients who developed symptomatic disease from prodromal stages.^39^ Therefore, targeting ICs could have therapeutic potentials in COVID‐19 patients, in particular those with compromised adaptive immune responses including Th1 responses and cytotoxic CD8^+^ T‐cell response. However, such interventions may only benefit patients with initial to intermediary disease states as T‐cell exhaustion in critical patients could be irreversible.^118^ Up to date, the effect of IC inhibitors (ICIs) on disease symptoms and clinical outcomes in COVID‐19 patients has not been evaluated. In the setting of other viral infections, the efficacy of anti‐PD‐1 (pembrolizumab) has been evaluated in a small cohort of patients with John Cunningham virus infection.^119^ Targeting PD‐1 and possibly other ICs in COVID‐19 patients could be beneficial in releasing the brake of T‐cell exhaustion to induce more potent and sustained antiviral responses mediated by effector T cells and cytotoxic CD8^+^ T cells, and the development of functional memory T cells for long‐term immunity.^89^, ^120^

Four clinical trials have been designed to assess the safety and therapeutic efficacy of anti‐PD‐1 monoclonal antibody (mAb) in patients with COVID‐19 (NCT04268537, NCT04333914, NCT04356508 and NCT04413838) (Table 1). However, findings from preclinical models showed that targeting PD‐1/PD‐L1 axis could result in excessive inflammation and tissue damage.^121^, ^122^ There is a possible risk of immune‐related adverse events (irAEs) and uncontrolled activation of immune cells associated with the use of ICIs, in particular those targeting PD‐1/PD‐L1, which rarely cause life‐threatening or fatal complications, such as myocarditis and pneumonitis.^123^

To avoid any potential risks of severe inflammation^124^ and obtain effective clinical outcomes, it would be ideal to combine anti‐PD‐1 mAb with other treatments, which minimize the immunopathology caused by cytokine storm, such as anti‐IL‐6 receptor mAb or anti‐IL‐1 receptor mAb.^81^, ^125^ Clinical trials have been planned to evaluate the safety and efficacy of these anti‐inflammatory treatments. Therefore, it is encouraging to examine the effect of combined therapy, anti‐PD‐1 with anti‐IL‐6R or anti‐IL‐1 receptor mAbs in COVID‐19 patients. To assess the potential benefits of suppressing the hyperinflammatory state on disease outcomes, clinical trials have been registered and planned to evaluate the safety and therapeutic efficacy of using IL‐6 and IL‐1 blockers or Bruton's tyrosine kinase (a key signalling pathway in macrophage activation and cytokine production) small molecule inhibitor in severe COVID‐19 patients.^9^, ^81^, ^125^, ^126^, ^127^ However, precautions are required during the assignment of therapeutic protocols, which simultaneously target PD‐1 and IL‐6 signalling pathways, and the time course of COVID‐19 infection is an important factor that should be considered and could predict the response to therapy. Early therapeutic interventions to block IL‐6 signalling during the early phase of disease, for example in patients with mild symptoms, may have detrimental effects and may result in insufficient adaptive and innate immune response required for viral clearance. On the other hand, late therapeutic interventions to block both PD‐1 and IL‐6 signalling pathways in ICU COVID‐19 patients and severe cases may have better clinical outcomes associated with T‐cell exhaustion reversal and reduced tissue damage and systemic inflammation.^39^, ^81^, ^128^ Overall, these approaches aim to stimulate antiviral immunity and prevent secondary complications of SARS‐CoV‐2 infection, which include sepsis and ARDS onset, concomitant with T‐cell depletion and elevated inflammatory cytokine release.

## FUTURE DIRECTIONS

Despite the devastating global impact of COVID‐19, there is no proven specific antiviral therapy in clinical use at present and treatment regimens mainly involve palliative therapies for treating comorbidities.^3^ COVID‐19 treatment requires a deep understanding of immune responses during the course of disease, in particular T‐cell responses, which exhibit a protective role at early stages of disease but could also contribute to the onset of fatal comorbidities. Appropriate control of SARS‐CoV‐2 infection and disease management require a timely therapeutic intervention to target inflammation and prevent disease worsening and secondary complications in severe cases. Early therapeutic interventions to block proinflammatory cytokines in patients with mild symptoms may have detrimental effects and result in insufficient immune response and impaired viral clearance. On the other hand, late therapeutic interventions to revert T‐cell exhaustion and ameliorate hyperinflammatory response in critical COVID‐19 patients may have better clinical outcomes.

Various therapeutic strategies and vaccines have been proposed and will be investigated in different clinical trials. These include T‐cell adoptive transfer therapy, viral vector‐, nucleic acid‐, protein‐ and DC‐based vaccines comprising of autologous DCs primed with specific viral antigens, and GM‐CSF could be useful to boost antiviral immunity and specific T‐cell responses. A potential self‐adjuvanting approach on vaccine production using multiple antigenic peptides is imperative to induce broad antiviral responses including adequate antibody production and T‐cell‐mediated immune responses against SARS‐CoV‐2. Additionally, cytokine therapies such as the administration of recombinant IL‐7 and low dose of IL‐2 could be beneficial in restoring T‐cell counts and expanding Tregs in COVID‐19 patients, especially in those with severe disease to control excessive inflammatory response. Furthermore, blocking cytokines to skew T‐cell induction towards Th1 responses via antibodies targeting cytokines such as IL‐10 and IL‐4 could result in favourable outcomes.^129^, ^130^ The blockade of TGF‐β, which could be released by T‐cell subsets, in particular Tregs, in COVID‐19 patients could also serve as future therapeutic approach to prevent excessive oedema, neutrophil recruitment to the lung and fibrosis in the lungs.^131^ Moreover, combined blockade of ICIs with anti‐inflammatory drugs could be beneficial in preventing the occurrence of potential risks associated with severe immune‐related adverse events.

Developing a TCR database using high‐throughput TCR sequencing on mild, severe, deceased and recovered COVID‐19 cohorts would be beneficial to quantify pathogen‐specific TCR repertoire with potential diagnostic/prognostic values. Additionally, studies focusing on SARS‐CoV‐2 reconstruction using genetic engineering platforms would be beneficial to understand the immune responses and downstream viral targets. Furthermore, identifying specific SARS‐CoV‐2 targets to develop antiviral agents and biomarkers to predict the clinical response to therapy would benefit disease management.

## AUTHOR CONTRIBUTIONS

ST, RS and VN wrote the article and prepared figures/table. RT assisted in writing – review and editing. EE conceived the concept, acquired funds, supervised and performed writing – review and editing.

## COMPETING INTEREST

The authors declare no conflicts of interest.

## ETHICAL APPROVAL

Not applicable.

## DATA SHARING STATEMENT

Data sharing not applicable – no new data generated.
